# Evolution of Human Respiratory Syncytial Virus (RSV) over Multiple Seasons in New South Wales, Australia

**DOI:** 10.3390/v10090476

**Published:** 2018-09-06

**Authors:** Francesca Di Giallonardo, Jen Kok, Marian Fernandez, Ian Carter, Jemma L. Geoghegan, Dominic E. Dwyer, Edward C. Holmes, John-Sebastian Eden

**Affiliations:** 1 Marie Bashir Institute for Infectious Diseases and Biosecurity, Charles Perkins Centre, School of Life and Environmental Sciences and Sydney Medical School, The University of Sydney, Sydney, NSW 2006, Australia; fdigiallonardo@kirby.unsw.edu.au (F.D.G.); edward.holmes@sydney.edu.au (E.C.H.); 2 The Kirby Institute, University of New South Wales, Randwick, NSW 2052, Australia; 3 Institute for Clinical Pathology and Medical Research, NSW Health Pathology, Westmead Hospital and University of Sydney, Sydney, NSW 2145, Australia; jen.kok@health.nsw.gov.au (J.K.); marian.fernandez@health.nsw.gov.au (M.F.); ian.carter@health.nsw.gov.au (I.C.); dominic.dwyer@sydney.edu.au (D.E.D.); 4 Department of Biological Sciences, Macquarie University, Sydney, NSW 2109, Australia; jemma.geoghegan@mq.edu.au; 5 Centre for Virus Research, Westmead Institute for Medical Research, Westmead, NSW 2145, Australia

**Keywords:** respiratory syncytial virus, phylogenetics, evolution, multi-year persistence

## Abstract

There is an ongoing global pandemic of human respiratory syncytial virus (RSV) infection that results in substantial annual morbidity and mortality. In Australia, RSV is a major cause of acute lower respiratory tract infections (ALRI). Nevertheless, little is known about the extent and origins of the genetic diversity of RSV in Australia, nor the factors that shape this diversity. We have conducted a genome-scale analysis of RSV infections in New South Wales (NSW). RSV genomes were successfully sequenced for 144 specimens collected between 2010–2016. Of these, 64 belonged to the RSVA and 80 to the RSVB subtype. Phylogenetic analysis revealed a wide diversity of RSV lineages within NSW and that both subtypes evolved rapidly in a strongly clock-like manner, with mean rates of approximately 6–8 × 10^−4^ nucleotide substitutions per site per year. There was only weak evidence for geographic clustering of sequences, indicative of fluid patterns of transmission within the infected population and no evidence of any clustering by patient age such that viruses in the same lineages circulate through the entire host population. Importantly, we show that both subtypes circulated concurrently in NSW with multiple introductions into the Australian population in each year and only limited evidence for multi-year persistence.

## 1. Introduction

Respiratory syncytial virus (RSV) is a major cause of acute respiratory tract infections (ARTI) in humans [1]. The burden of RSV disease is greatest in specific vulnerable populations, including young children and the elderly, particularly those with pre-existing medical comorbidities or who are immunocompromised. Outbreaks in hospitals and closed environments, such as aged care facilities, have also been documented [2]. Importantly, the annual global hospitalization rate of RSV infection in young children is nearly 10% and is associated with approximately 59,600 deaths [3,4]. The health and economic burden of RSV in young children surpasses that of the influenza virus with total annual direct health care costs estimated to be between $24–50 million [5]. In Australia, Indigenous children living in remote communities also experience a high prevalence of RSV, particularly in comparison to non-Indigenous groups [6,7]. Morbidity and mortality are also high in elderly adults, with approximately 14,000 deaths annually due to RSV in the USA (Centre for Disease Control [8]).

While the factors that contribute to the prevalence of RSV are yet to be fully defined, immunity, re-infection rates, and climate may play a role. For example, laboratory reports highlight the seasonality in temperate regions in Australia, with a peak in RSV activity typically occurring in the early winter (May/June) period and preceding the seasonal peak in influenza virus (New South Wales Health, Influenza Monthly Surveillance Reports). Climate and rainfall differences in the tropical north of Australia are also likely to be important drivers of RSV disease patterns, resulting in a seasonality distinct from that observed in temperate regions, with a correlation between peak RSV and peak rainfall levels around January [6].

RSV is a negative-sense single-stranded RNA virus (family *Pneumoviridae*) with a 15 kb genome that encodes 10 proteins [9]. Two distinct antigenic subgroups have been identified, subtypes A and B (RSVA and RSVB, respectively) that show clear phylogenetic divergence [10,11]. The glycoprotein (G), responsible for attachment to the host cell, exhibits the greatest genetic diversity within and between the subtypes [11]. This is thought to reflect strong immune pressure and the subsequent generation of escape variants in a process analogous to antigenic drift in the hemagglutinin (HA) protein of the influenza A virus [12,13]. Hence, reinfection with RSV is commonplace [14,15]. There are currently no effective vaccines against RSV, although a number of novel vaccines are entering clinical trials [16]. Similarly, there are new antivirals targeting the viral polymerase and a fusion protein that are in clinical trials [17].

Despite the clinical significance and the burden of RSV infection worldwide, we lack an understanding of the patterns of virus emergence, evolution and spread. Phylogenetic studies of global RSV evolution are compromised due to the limited availability of whole genome sequence data and strongly asynchronous sampling in time and space. Most evolutionary analyses have focused on the *G gene* because of its high genetic diversity and utility as a phylogenetic marker. The *G gene* is also characterized by premature stop codons in the case of RSVB [18,19] and duplications in the *G gene* are described in both subtypes [20,21]. Less is known about the genome-scale evolution of RSV [21,22,23,24,25], although this is necessary for defining fine-scale phylodynamic and epidemiological processes that may assist in targeted interventions including vaccine design and implementation [20,22,26].

There have been several studies of RSV evolution in specific geographic regions, including South Africa [25], the Netherlands [23], Argentina [21], Italy [24], and Kenya [22]. These studies highlight the global distribution of predominant RSV variants during each season, alongside the establishment and co-circulation of local endemic sub-lineages. There is, however, limited data exploring the genetic diversity of RSV in Australia. Similarly, the evolution and spread of RSV within specific communities, and hence how long individual lineages of RSV are able to persist in single populations, is not well understood. To address these issues, we provide the first large, genome-scale analysis of RSV in Australia, focusing on infections identified through a major clinical diagnostic laboratory that services a population of over 1.57 million people. In particular, we sought to determine the extent and pattern of genetic diversity circulating within the culturally diverse region of western Sydney as well as the rural region of western New South Wales (NSW), how this relates to the global diversity of the virus, what epidemiological factors act to shape genetic diversity at the local level, and to what extent RSV transmission persists between seasonal outbreaks.

## 2. Materials and Methods

### 2.1. Ethics

This study was approved by local ethics and governance committees (LNR/17/WMEAD/128 and SSA/17/WMEAD/129 on 13 April 2017). Samples were de-identified with basic demographic information collected including age, sex and location (city, region, hospital).

### 2.2. Sample Collection

This study utilized residual RSV-positive specimens collected for routine diagnostic testing at the Institute of Clinical Pathology and Medical Research (ICPMR), Westmead Hospital, NSW, Australia between May 2010 and December 2016. Viral nucleic acid that was previously extracted (NucliSENS^®^ easyMAG^®^, bioMérieux) during routine diagnostic testing was stored at −80 °C prior to the commencement of this study. 

### 2.3. Whole Genome Sequencing

We employed an overlapping RT-PCR strategy to amplify viral genomes (four ~4 kb amplicons), targeting both RSVA and RSVB subtypes using previously published primers [27]. Briefly, viral RNA was first reverse transcribed using a pool of the four forward primers (RSVS1_01F, RSVS2_3905F, RSVS3_7215F and RSVS4_10959F) and the SuperScript™ IV cDNA synthesis system (Invitrogen, Thermo Fisher Scientific, Waltham, MA, USA). The resultant cDNA was then split across four parallel PCR reactions to amplify the genome using Platinum SuperFi (Invitrogen, Carlsbad, CA, USA). For the successfully amplified samples, the four amplicons were pooled equally and then prepared as libraries using Nextera XT before MiSeq (Illumina, San Diego, CA, USA) sequencing. Each sequencing run contained between 41 and 60 indexed samples, which generated at least 2000X per base coverage per genome. Raw sequence reads were quality trimmed with Trimmomatic [28] and then de novo assembled using Trinity [29] and SPAdes [30]. The trimmed reads were re-mapped to draft RSV genome contigs with BowTie2 [31]. The mapping alignment quality was checked manually particularly around known *G gene* duplications before extracting the final majority consensus genome sequence for each sample.

### 2.4. Data Availability

All genome sequences generated in this study are available on NCBI GenBank with accession numbers MH760588 to MH760731, as shown in supplementary Table S1.

### 2.5. Phylodynamic Analysis

To place our sample set into a global context, complete and near complete genome sequences of RSVA and RSVB were obtained from GenBank. Sequences with no geographic association or sampling date were excluded. RSVA and RSVB sequences were aligned separately using the multiple-sequence alignment tool, MAFFT, using the L-INS-I algorithm followed by a visual inspection [32]. Sequences resulting from passaging experiments, or potential recombinants identified using RDP4 [33] were removed from the alignment. After this data pruning, the final data set consisted of 849 RSVA of 15,062-nt length and 500 RSVB genome sequences of 15,033-nt length (Table S1). Intergenic regions were removed for phylogenetic tree estimates as they contained single nucleotide insertions and deletions. 

Maximum likelihood (ML) trees were estimated in RAxML [34,35] employing a GTR gamma (Γ) nucleotide substitution model and 1000 bootstrap replications. To determine the extent of the temporal structure in the data, a root-to-tip regression of genetic distance against year of sampling was performed using TempEst v.1.5 utilizing the separate ML trees for RSVA and RSVB [36]. As both RSVA and RSVB exhibited a strong temporal structure (i.e., clock-like evolution; see Results), we estimated their evolutionary rates more accurately using the Bayesian Markov chain Monte Carlo (MCMC) method implemented in BEAST v1.8.2 [37], using an HKY + gamma (Γ) substitution model. A strict clock was used for the evolutionary rate estimates and a constant population size was implemented as a tree prior (although no significant differences in evolutionary rate were observed when we compared the rate estimates from the uncorrelated log-normal relaxed clock to those from the strict clock). All analyses were run for at least 100 million steps and sampling every 10,000 steps to ensure convergence of all parameters. The first 10% of the posterior was removed as burn-in. Mean rates and 95% highest posterior density (HPD) were compared and values with HDP non-overlapping with mean rate values were significantly different. However, because we were unable to achieve consistent statistical convergence for the global data set, evolutionary rates were instead estimated by implementing a least-square dating algorithm (LSD) which is suitable for large data sets [38,39]. To obtain significance, 1000 parametric bootstraps were conducted on the branch lengths. 

### 2.6. Phylogenetic Analysis of Clustering Patterns

**Figure 1:**
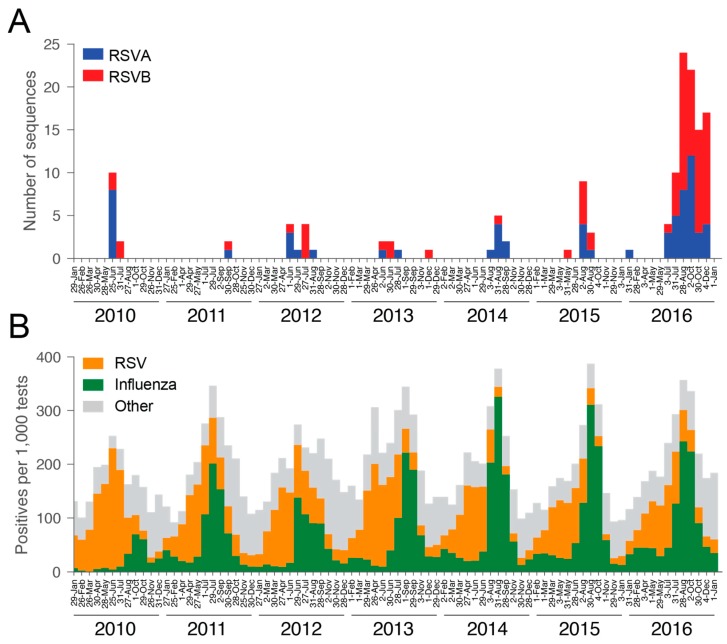
Incidence of respiratory syncytial virus (RSV) in New South Wales (NSW). (**A**) Number of RSV genome sequences per four-week period: RSVA = blue, RSVB = red. (**B**) Number of positive samples per 1000 specimens reported across NSW per four-week period: RSV = orange, Influenza = green, Other, including parainfluenza virus, adenovirus, and human metapneumovirus = grey.

We used a phylogenetic approach to determine whether there was more clustering by geography and age within the NSW RSV data set than might be expected from chance alone. Specifically, Bayesian posterior trees were used for evaluating geographic and age structure in the RSVA and RSVB trees using the Bayesian Tip-association Significance (BaTS) program; which compares parsimony score (PS), association index (AI), and maximum clade size (MC) statistics [40]. Estimates were repeated 1000 times to infer significance. The traits investigated were age, the hospital facility where patients first presented, and state electorate. Due to the large sampling biases in the data, with few sequences obtained from most years (Figure 1A), this analysis was only performed on the samples from 2016 as it was by far the most densely sampled year (RSVA = 36 sequences, RSVB = 57).

## 3. Results and Discussion

### 3.1. Demographic Characteristics of RSV in NSW

**Figure 2:**
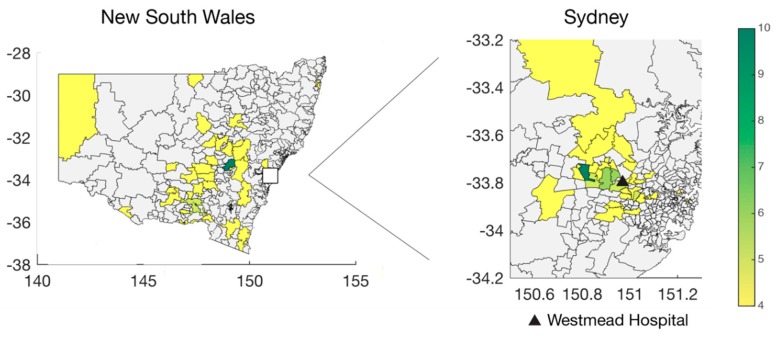
Geographic distribution of respiratory syncytial virus in NSW. Number of sequences per postcode in NSW (**left**) and greater Westmead area (**right**). The location for Westmead Hospital is indicated.

We attempted whole genome sequencing on 241 archived RSV-positive viral nucleic acid extracts held and tested by ICPMR. Virus genomes were successfully sequenced for 144 specimens, of which 64 belonged to RSVA and 80 to RSVB. The number of samples was skewed with the majority collected in 2016, comprising 36 RSVA and 57 RSVB sequences, respectively (Figure 1A). Despite this limited sampling, it is evident that RSVA and RSVB co-circulated in every season, which is consistent with other molecular epidemiological studies [41,42,43,44]. A distinct seasonality was apparent with peaks typically occurring in the early winter period (May to July) (Figure 1A). This pattern is consistent with aggregated data from state-wide testing across NSW for RSV, influenza, and other respiratory viruses (Figure 1B) [45]. For RSVA, 54.7% of the samples were derived from female patients, while 43.8% of RSVB samples were from female patients (Table 1). Forty-five per cent of RSVA sequences were obtained from patients under the age of two, and 27% from patients greater than 65 years of age. These numbers were slightly lower for RSVB, with 43% being infants and only 20% older patients. While all testing and sequencing were performed at Westmead Hospital, the cases were not limited to the locations surrounding the hospital, but also included samples from western and north-western rural NSW and hence some distance from metropolitan Sydney (Figure 2). As a consequence, the samples collected here will be referred to as from NSW. The electorates with the most sequences sampled were Orange (*n* = 10) and Mount Druitt (*n* = 11). Despite the wide geographic range of sampling, coverage was low. Hence, sequences were sampled only from a small number of geographic locations. 

### 3.2. Evolutionary History of RSV and Spread within NSW

**Figure 3:**
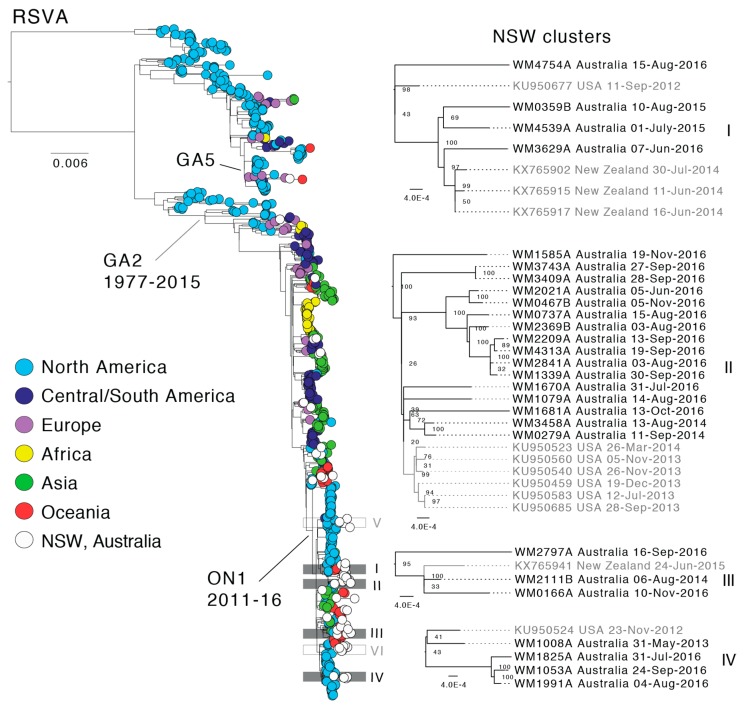
Global phylogeny of respiratory syncytial virus subtype A (RSVA) and local clustering within NSW. The maximum likelihood tree shown was estimated using complete RSV genome sequences. Known genotypes and the sequence time range is indicated. The tree was rooted using an RSVB outgroup. Tree tips are colored according to the geographic region and sequences from this study are shown in white. Local clusters comprising NSW sequences are marked within the global tree and the four clusters with potential multi-season transmission events are colored grey and enlarged on the right side. Node supports are indicated and branch lengths are scaled according to the number of substitutions per site.

**Figure 4:**
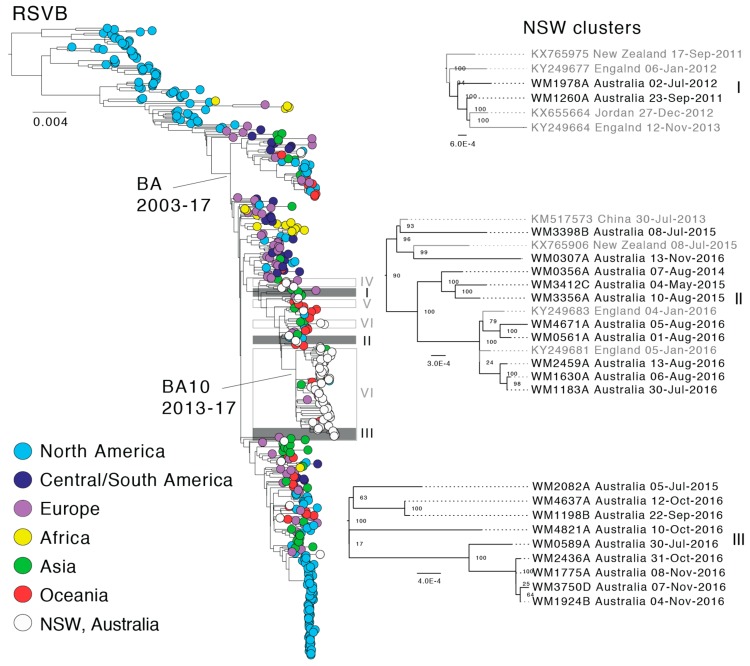
Global phylogeny of respiratory syncytial virus subtype B (RSVB) and local clustering within NSW. The maximum likelihood tree shown was estimated using complete RSV genome sequences. The tree was rooted using RSVA as an outgroup and the BA and BA10 genotypes are marked. Tip colors represent the sampling location and sequences from this study are shown in white. Local clusters comprising NSW sequences are marked within the global tree and the three clusters with potential multi-season transmission events are colored grey and enlarged on the right side. Node supports are indicated and branch lengths are scaled according to the number of substitutions per site.

To place our Australian RSV strains in the context of global RSV diversity, we performed an evolutionary analysis using our genome sequences with global reference genomes sourced from GenBank (Table S1). These sequences were sampled from 21 different countries across multiple continents and spanned a time-span of 40 years ranging from 1977 to 2017. The final combined data set consisted of 849 and 500 RSVA and RSVB genomes, respectively. While 21 countries were represented in the data, there was a clear over-representation (*n* = 628) of viral genomes from the USA, which comprised 46% of the data in this study. Other relatively well represented countries were Peru (*n* = 122), the Netherlands (*n* = 61), Kenya (*n* = 61), Jordan (*n* = 85), Viet Nam (*n* = 53), New Zealand (*n* = 92), and the sequences sampled here in NSW (*n* = 144). The extensive sampling biases precluded detailed phylogeographic analyses. To simplify the geographic distribution analysis, sequences were grouped according to their continent of sampling (Figure 3 and Figure 4).

In both the RSVA and RSVB phylogenies, the earliest described RSV genomes were derived solely from North America, and it is difficult to comment on the global distribution, diversity and genetic sources until the early-mid 2000s when sampling became more evenly distributed. Since this time, the global RSVA phylogeny has been dominated by viruses of the GA2 lineage, and more recently, the ON1 sub-lineage (Figure 3), which is defined by a 72-nt duplication in the *G gene* [21]. In the global RSVB phylogeny, three sub-lineages of BA viruses have co-circulated, with the exception of the most recent samples in which BA10 viruses appear to be dominant; although this could again reflect sampling biases (Figure 4). In both phylogenies, distinct geographical clusters are clearly visible. These sequences, sampled often from the same country and within a short time frame, are seemingly indicative of local outbreaks following the importation of a globally predominant variant. For example, there is a distinct cluster (Figure 3, yellow) of sequences from Kenya, collected during 2010–2012, and a large cluster (Figure 4, light blue) of sequences sampled in Tennessee (USA) in 2013–2014.

The sequences from this study fell across the global RSVA and RSVB phylogenies, indicative of multiple entries of the virus into NSW, both within and between individual RSV seasons (Figure 3 and Figure 4). We defined NSW-specific sequence clusters as nodes with a majority of NSW sequences compared to the background of global sequences. Accordingly, we identified six and seven such clusters for RSVA and RSVB, respectively. For RSVA, all six NSW clusters were from the ON1 genotype, five of which harbored sequences from 2016 (Figure 3). Due to the bias toward 2016, it was difficult to determine the extent of off-season RSV transmission (i.e., ‘over-summering’). However, some evidence for the persistence of virus within Australia between RSV seasons was observed in 2015 and 2016 (Figure 3, clusters I and II) and perhaps over multiple seasons (Figure 3, clusters II, III, and IV). However, the genetic distances between the sequences are large and node support is low so that the clustering of sequences from different years is perhaps more likely to be due to limited sampling rather than actual virus persistence. 

In the case of RSVB, six sequence pairs in individual clusters and the large BA10 genotype cluster were identified (Figure 4). Potential multiple entries might have occurred in 2012 and 2016 for the BA genotype, excluding the BA10 cluster, although this inference is again based on only a small number of sequences. Interestingly, there is some evidence for multi-season persistence from 2011 to 2012 and 2015 to 2016, although this will clearly need to be confirmed with larger data sets (Figure 4, clusters I and II). Similarly, within the BA10 genotype there is some evidence of multi-year persistence from 2015 to 2016 (Figure 4, cluster III, WM2082A). The BA10 genotype contains Australian sequences sampled between 2013 and 2016 and sequences from the USA, China, New Zealand, England, and Japan, with the latter the most recent sequence sampled in 2017. Thus, the BA10 genotype likely represents the most recent global circulating RSVB variant.

### 3.3. Geographic and Age Structure of RSV Infections in NSW

For the most comprehensive sampled year, 2016, we assessed the extent to which the phylogenetic structure in the data reflected patient age or geographic structure. Accordingly, the facility (hospital or clinic) where the patient first presented, patient electorate, and patient age were mapped across the tree (Table S2). Thirteen different facilities were associated with RSVA, although six facilities were associated with one sequence only. Similarly, RSVB was represented by 18 facilities, nine of which were represented by one sequence only. Nevertheless, one facility, Young Hospital (located in rural NSW) contained four RSVA sequences and exhibited significant clustering with a *p*-value of 0.001 (Table S2, RSVA). These sequences were sampled in August and September and most likely represent a distinct local outbreak as genetic diversity was low (Figure 3, cluster II, WM1339A, WM2841A, WM2209A & WM4313A). All four sequences were sampled from patients residing in the Cootamundra electorate, which is also the electorate with the lowest *p*-value (0.002). The significant clustering for facility and electorate is also supported by the low parsimony score (PS) and association index (AI) values, i.e., 0.00 and 0.006 for facility and electorate, respectively. Surprisingly, no significant clustering was observed in the case of RSVB despite different geographic locations and age categories being well represented in the data (Table S2). Notably, however, both the AI and PS statistics are highly conservative [46], as the null model assumes complete panmixis, and thus weak significance may, in fact, indicate relatively frequent virus movement. This is supported by the observation that the best-sampled localities often exhibited the least geographic clustering, arguing against in situ transmission. For example, Mount Druitt Hospital and Westmead Hospital were the best-sampled facilities (13 and 11 sequences for RSVA and RSVB, respectively) but exhibited non-significant geographic clustering (*p* = 0.730, and 1.00, respectively). Finally, we also investigated the extent of phylogenetic clustering by patient age group, particularly as RSV mainly infects infants. Notably, none of the age groups showed significant clustering in RSVA or RSVB (Table S2). Hence, these data indicate that the same virus lineages were able to infect and circulate within multiple age groups.

### 3.4. Evolutionary Dynamics of RSV

**Figure 5:**
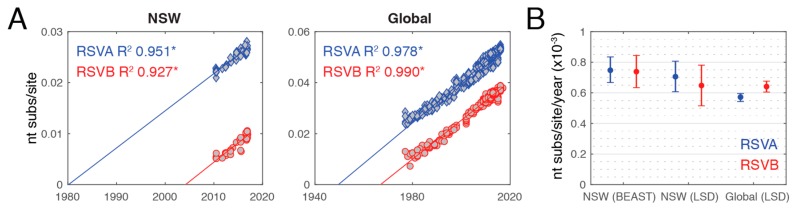
**Evolutionary rates in respiratory syncytial virus (RSV).** (**A**) Linear regressions of root-to-tip genetic distances against sampling date based on maximum likelihood trees. The R^2^ value for each regression is indicated and corresponding *p* values <0.001 are indicated with an asterisk. Sequences from NSW in this study are shown on the left and global sequences on the right. (**B**) Estimates of the nucleotide substitution rate per site per year are shown for sequences from NSW (BEAST and least-square dating algorithm (LSD) estimates) and globally (LSD estimates only). Rates are shown as mean values (circles) and the 95% highest posterior density (HPD) and confidence intervals for BEAST and LSD, respectively (error bar). (RSVA: blue; RSVB: red).

Previous studies have reported a difference in evolutionary rates between the two subtypes, particularly that the *G gene* had significantly higher rates in RSVB than RSVA [20,47]. As the global RSV data is highly biased in time and space, we examined evolutionary dynamics at both the global scale and within the NSW sequences alone. To assess the extent of clock-like structure in the data, we first performed a simple regression of genome-scale root-to-tip genetic distances against year of sampling with TempEst v.1.5 [36] using the RSVA and RSVB ML trees. This provided evidence for a very strong molecular clock signal, with R^2^ values of 0.951 and 0.927 for RSVA and RSVB, respectively, for the NSW sequences, and 0.978 and 0.990 for the global sequences (Figure 5A). Under this regression method, the mean rates of nucleotide substitution were also very similar at 7.97 and 7.62 × 10^−4^ substitutions per site per year (subs/site/year) for global RSVA and RSVB, respectively, and 7.29 and 7.56 × 10^−4^ subs/site/year for RSVA and RSVB sampled in NSW in this study, respectively (Figure 5A).

Given this strong clock-like structure, we investigated the evolutionary rates more carefully using the Bayesian Markov chain Monte Carlo (MCMC) method implemented in BEAST for the NSW data set and the least square dating (LSD) method for the global data set. In the case of the NSW data, the mean evolutionary rates were 7.06 × 10^−4^ (confidence interval 6.67–8.34 × 10^−4^) and 6.48 × 10^−4^ (confidence interval 5.15–7.81 × 10^−4^) subs/site/year for RSVA and RSVB, respectively, using LSD and 7.48 × 10^−4^ (95% HPD 6.67–8.34 × 10^−4^) and 7.39 × 10^−4^ (95% HPD 6.34–8.45 × 10^−4^) subs/site/year for RSVA and RSVB, respectively, using BEAST (Figure 5B). Hence, there was no significant difference in rate between RSVA and RSVB. For the global data set, the LSD rate estimates were 5.72 × 10^−4^ (confidence interval 5.43–5.95 × 10^−4^) and 6.41 × 10^−4^ (confidence interval 6.02–6.78 × 10^−4^) subs/site/year for RSVA and RSVB, respectively. These rates are within the range reported previously for paramyxoviruses [48] and other single-stranded RNA viruses [49]. Notably, the substitution rates for the global data set were significantly different between RSVA and RSVB, and RSVB exhibited a higher rate, as previously described [20]. These global rates were also consistently lower than those observed within NSW (for both BEAST and LSD) and there was no difference between RSVA and RSVB in the NSW data set. This difference between the local (NSW) and global rates may reflect the fact that the NSW data were sampled more recently and that rates were elevated towards the present because of time-dependent evolution; itself reflecting incomplete purifying selection that is commonly observed in RNA viruses [50,51].

## 4. Conclusions

We report a wide diversity of RSV lineages co-circulating in a small geographic region, reflecting a combination of continual virus entry and some sustained in situ transmission within NSW. Despite these fine-scale epidemiological insights, this study also highlighted the highly biased global sampling of RSV that hinders extensive analysis of the global distribution and transmission dynamics of RSV. We stress that increased targeted surveillance with more extensive virus sampling, particularly during suspected outbreaks, is essential to improve both our understanding of RSV ecology and evolution, and assist with vaccine design.

## Figures and Tables

**Table 1:** Demographic of the patient data used in this study. Ratios for gender and age categories are shown for respiratory syncytial virus (RSV) A and B. Total numbers are shown in brackets.

Age Category	RSVA (*n* = 64)		RSVB (*n* = 80)	
	Male	Female	Male	Female
All	0.453 (29)	0.547 (37)	0.550 (44)	0.438 (35)
6 months or younger	0.094 (6)	0.078 (5)	0.163 (13)	0.075 (6)
7 months to 1 year	0.078 (5)	0.125 (8)	0.063 (5)	0.038 (3)
1–2 years	0.031 (2)	0.047 (3)	0.025 (2)	0.063 (5)
2–5 years	0.016 (1)	0.031 (2)	0.025 (2)	0.038 (3)
6–15 years	0.016 (1)	0.016 (1)	0.000 (0)	0.000 (0)
16–25 years	0.016 (1)	0.016 (1)	0.025 (2)	0.013 (1)
26–49 years	0.047 (3)	0.031 (2)	0.063 (5)	0.063 (5)
50–65 years	0.016 (1)	0.078 (5)	0.088 (7)	0.050 (4)
66 years or older	0.141 (9)	0.125 (8)	0.100 (8)	0.100 (8)

## Supplementary Materials

The following are available online at http://www.mdpi.com/1999-4915/10/9/476/s1, Table S1: Accession numbers; Table S2: Phylogeny-trait association test for RSV in Australia.
