# Supplementary material for: Evolution of Human Respiratory Syncytial Virus (RSV) over Multiple Seasons in New South Wales, Australia
Source: Viruses. 2018 Sep 6;10(9):476. doi: 10.3390/v10090476 (PMC6164696; doi:10.3390/v10090476)
Supplement: Supplementary file 1 [file viruses-10-00476-s001.zip › viruses-351551-supplementary/Supp/Supplementary_Table_S2.docx]

| **Supplementary Table 2.** Phylogeny-trait association test for RSV in Australia. | | | | | | |
| --- | --- | --- | --- | --- | --- | --- |
| **RSVA** | | |  | **RSVB** | | |
| *Facility* | n | *p* value |  | *Facility* | n | *p* value |
| Auburn Hospital | 2 | 1.000 |  | Auburn Hospital | 5 | 1.000 |
| Blacktown Hospital | 1 | 1.000 |  | Bega Hospital | 5 | 1.000 |
| Broken Hill Base Hospital | 1 | 1.000 |  | Blacktown Hospital | 2 | 1.000 |
| Cooma Hospital | 1 | 1.000 |  | Broken Hill Base Hospital | 1 | 1.000 |
| Deniliquin Hospital | 1 | 1.000 |  | Coolamon Multi-purpose Service | 1 | 1.000 |
| Dubbo Base Hospital | 3 | 0.040 |  | Dubbo Base Hospital | 10 | 0.274 |
| Leeton Hospital | 2 | 0.013 |  | Forbes District Hospital | 1 | 1.000 |
| Mount Druitt Hospital | 13 | 0.730 |  | Goulburn Hospital | 3 | 1.000 |
| Mudgee Hospital | 1 | 1.000 |  | Grenfell District Hospital | 1 | 1.000 |
| Orange Base Hospital | 2 | 1.000 |  | Mount Druitt Hospital | 5 | 1.000 |
| Wagga Wagga Base Hospital | 1 | 1.000 |  | Narrandera District Hospital | 1 | 1.000 |
| Westmead Hospital | 4 | 0.087 |  | Orange Base Hospital | 3 | 1.000 |
| *Young Hospital* | *4* | *0.001* |  | Parkes District Hospital | 1 | 1.000 |
|  |  |  |  | Temora and District Hospital | 1 | 1.000 |
|  |  |  |  | Tumbarumba Multi-purpose Service | 1 | 1.000 |
|  |  |  |  | Tumut District Health Service | 1 | 1.000 |
|  |  |  |  | Wagga Wagga Base Hospital | 4 | 1.000 |
|  |  |  |  | Westmead Hospital | 11 | 1.000 |
|  |  |  |  |  |  |  |
| *Electorate* |  |  |  | *Electorate* |  |  |
| Auburn | 2 | 1.000 |  | Albury | 3 | 1.000 |
| Blacktown | 1 | 1.000 |  | Auburn | 5 | 1.000 |
| Cabramatta | 1 | 1.000 |  | Barwon | 6 | 1.000 |
| Clarence | 1 | 1.000 |  | Bega | 4 | 1.000 |
| Cootamundra | 6 | 0.002 |  | Cootamundra | 5 | 1.000 |
| Dubbo | 3 | 1.000 |  | Dubbo | 4 | 1.000 |
| Londonderry | 2 | 1.000 |  | Goulburn | 3 | 1.000 |
| Monaro | 1 | 1.000 |  | Granville | 1 | 1.000 |
| Mount Druitt | 8 | 0.336 |  | Hawkesbury | 1 | 1.000 |
| Murray | 1 | 1.000 |  | Londonderry | 3 | 1.000 |
| Orange | 2 | 1.000 |  | Mount Druitt | 2 | 1.000 |
| Prospect | 2 | 1.000 |  | Orange | 6 | 1.000 |
| Riverstone | 2 | 1.000 |  | Parramatta | 2 | 1.000 |
| Seven Hills | 2 | 1.000 |  | Prospect | 1 | 1.000 |
| Wagga Wagga | 1 | 1.000 |  | Riverstone | 2 | 1.000 |
|  |  |  |  | Seven Hills | 4 | 1.000 |
|  |  |  |  | Strathfield | 1 | 1.000 |
|  |  |  |  | Wagga Wagga | 3 | 1.000 |
|  |  |  |  |  |  |  |
| *Age Category* |  |  |  | *Age Category* |  |  |
| 6 months or younger | 8 | 1.000 |  | 6 months or younger | 17 | 0.165 |
| 7 months to 1 year | 10 | 1.000 |  | 7 months to 1 year | 6 | 1.000 |
| 1 - 2 years | 4 | 1.000 |  | 1 - 2 years | 7 | 1.000 |
| 2 - 5 years | 3 | 1.000 |  | 2 - 5 years | 4 | 1.000 |
| 6 - 16 years | 2 | 1.000 |  | 16 - 25 years | 2 | 1.000 |
| 50 - 65 years | 4 | 1.000 |  | 25 - 49 years | 5 | 1.000 |
| 66 years or older | 5 | 0.128 |  | 50 - 65 years | 5 | 1.000 |
|  |  |  |  | 66 years or older | 11 | 1.000 |

*Facility* AI 0.000 PS 0.000 *Facility* AI 0.659 PS 0.623

*Electorate* AI 0.006 PS 0.006 *Electorate* AI 0.917 PS 0.958

*Age* AI 0.777 PS 0.834 *Age* AI 0.582 PS 0.470
